# Reactive arthritis following COVID-19 current evidence, diagnosis, and management strategies

**DOI:** 10.1186/s13018-023-03651-6

**Published:** 2023-03-15

**Authors:** Filippo Migliorini, Andreas Bell, Raju Vaishya, Jörg Eschweiler, Frank Hildebrand, Nicola Maffulli

**Affiliations:** 1 Department of Orthopaedic, Trauma, and Reconstructive Surgery, RWTH University Hospital, Pauwelsstraße 30, 52074 Aachen, Germany; 2 Department of Orthopaedic and Trauma Surgery, Eifelklinik St. Brigida, 52152 Simmerath, Germany; 3 Department of Orthopaedics, Indraprastha Apollo Hospitals Institutes of Orthopaedics, New Delhi, India; 4 Department of Medicine, Surgery and Dentistry, University of Salerno, 84081 Baronissi, SA Italy; 5 Faculty of Medicine, School of Pharmacy and Bioengineering, Keele University, Stoke-on-Trent, ST4 7QB England; 6 Barts and the London School of Medicine and Dentistry, Centre for Sports and Exercise Medicine, Mile End Hospital, Queen Mary University of London, London, E1 4DG England

**Keywords:** COVID-19, Reactive arthritis, Immunity

## Abstract

**Background:**

Immune-mediated conditions associated to Corona Virus Disease-19 (COVID-19) have been reported, including vasculitis, antiphospholipid antibody syndrome, myositis, and lupus. Emerging studies have reported the potential occurrence of reactive arthritis in patients previously infected with COVID-19. This systematic review summarised the current evidence on the occurrence of reactive arthritis in patients previously infected by COVID-19.

**Methods:**

This study was conducted according to the 2020 PRISMA guidelines. All the clinical investigations describing the occurrence of reactive arthritis following COVID-19 were accessed. In September 2022, the following databases were accessed: PubMed, Web of Science, Google Scholar, Embase. The generalities of the study were extracted: author, year and journal of publication, country of the main author, study design, sample size, mean age, number of women, main results of the study. The following data on COVID-19 severity and management were retrieved: type of treatment, hospitalization regimes (inpatient or outpatient), admission to the intensive care unit, need of mechanical ventilation, pharmacological management. The following data on reactive arthritis were collected: time elapsed between COVID-19 infection to the onset of reactive arthritis symptoms (days), pharmacological management, type of arthritis (mono- or bilateral, mono- or polyarticular), extra-articular manifestations, presence of tenosynovitis or enthesitis, synovial examination at microscopic polarised light, imaging (radiography, magnetic resonance, sonography), clinical examination, laboratory findings.

**Results:**

Data from 27 case reports (54 patients) were retrieved, with a mean age of 49.8 ± 14.5 years. 54% (29 of 54 patients) were women. The mean time span between COVID-19 infection and the occurrence of reactive arthritis symptoms was 22.3 ± 10.7 days. Between studies diagnosis and management of reactive arthritis were heterogeneous. Symptoms resolved within few days in all studies considered. At last follow-up, all patients were minimally symptomatic or asymptomatic, and no additional therapy or attentions were required by any patient.

**Conclusion:**

Poor evidence suggests that COVID-19 could target the musculoskeletal system causing reactive arthritis at its post infectious stage. COVID-19 can act as a causative agent or as a trigger for development of reactive arthritis even without presence of antibodies of rheumatological disorders. Treating physicians should have a high index of suspicion while treating post infectious COVID-19 patient with arthralgia.

**Level of evidence:**

Level IV, systematic review.

## Introduction

Immune-mediated conditions associated to Corona Virus Disease-19 (COVID-19) infection have been reported, including vasculitis, antiphospholipid antibody syndrome, myositis, and lupus [1, 29]. The underlying immune mechanisms behind the occurrence of immune-mediated manifestations following COVID-19 deserve further investigation. COVID-19 could induce transient immunosuppression [20], which may induce to immune-mediated innate response, with a marked elevation of IL-2, IL-2R, IL-6, IL-7, IL-8 IL-10, IP10, MIP1A, and TNF-α [7, 9, 20, 28, 40, 50]. These interleukins are key in the pathogenesis of psoriasis and psoriatic arthritis [6, 34, 48]. Hence, elevated levels of Interleukin-17 in serum have been observed also in patients with Middle East respiratory syndrome, whereas patients with COVID-19 demonstrated elevated circulating Th17 cells [44].

Emerging studies have reported the potential occurrence of reactive arthritis in patients previously infected with COVID-19. A syndrome consistent with reactive arthritis has been described following HIV, Dengue, Chickungunya and Parvo virus B19 [33, 38, 39, 52]. An extra-articular infection, typically sexually transmitted or gastro intestinal, may trigger reactive arthritis [56]. Common pathogens are Shigella, Chlamydia Trichomatis, Streptococcus, Yersinia, Salmonella and Compylobacter species [5, 31]. However, reactive arthritis may happen also following viral infections [3, 39].

This systematic review summarises the current evidence on the occurrence of reactive arthritis in patients previously infected by COVID-19.

## Methods

### Eligibility criteria

All the clinical investigations describing the occurrence of reactive arthritis following COVID-19 were accessed. According to the author´ language capabilities, articles in English, German, Italian, French and Spanish were eligible. Studies with level I–IV of evidence, according to Oxford Centre of Evidence-Based Medicine [27], were considered. Reviews, animals, in vitro, biomechanics, computational, and cadaveric studies were not eligible. Only clinical investigation which reported reactive arthritis clinically defined by signs of joint inflammation (dolor, rubor, calor, tumour, functio lesa, either alone or in combination) or by imaging modalities (ultrasounds and/or magnetic resonance) were eligible.

### Search strategy

This study was conducted according to the 2020 Preferred Reporting Items for Systematic Reviews and Meta-Analyses (PRISMA) [45]. The following algorithm was preliminary pointed out:

- *Problem* reactive arthritis following COVID;
- *Intervention* diagnosis and management;
- *Outcome* clinical outcome.

In September 2022, the following databases were accessed: PubMed, Web of Science, Google Scholar, Embase. No time constrain was set for the search. The following matrix of keywords were used in each database to accomplish the search using the Boolean operator AND/OR: (*COVID-19* OR *COVID* OR *SARS-CoV2* OR *pandemic*) AND (*reactive*) AND (*osteoarthritis* OR *arthritis* OR *knee pain* OR *ankle pain* OR *spine pain* OR *shoulder pain* OR *hand pain*). No additional filters were used in the databases search.

### Selection and data collection

Two authors (F. M. and H. S.) independently performed the database search. All the resulting titles were screened by hand and, if suitable, the abstract was accessed. The full-text of the abstracts which matched the topic were accessed. If the full-text was not accessible or available, the article was not considered for inclusion. A cross reference of the bibliography of the full-text articles was also performed for inclusion. Disagreements were debated and mutually solved by the authors. In case of further disagreements, a third author (F.H.) took the final decision.

### Data items

Two authors (F.M. and N.M.) independently performed data extraction. The generalities of the study were extracted: author, year and journal of publication, country of the main author, study design, sample size, mean age, number of women, main results of the study. The following data on COVID-19 severity and management were retrieved: type of treatment, hospitalization regimes (inpatient or outpatient), admission to the intensive care unit (ICU), need of mechanical ventilation, pharmacological management. The following data on reactive arthritis were collected: time elapsed between COVID-19 infection to reactive arthritis symptoms (days), pharmacological management, type of arthritis (mono- or bilateral, mono- or polyarticular), extra-articular manifestations, presence of tenosynovitis or enthesitis, synovial examination at microscopic polarised light, imaging (radiography, magnetic resonance, sonography), clinical examination, laboratory findings.

### Synthesis methods

The statistical analysis was performed by the main author (F.M.) using the software IBM SPSS (version 25). The arithmetic mean and standard deviation were used for continuous variables.

## Results

**Fig. 1:**
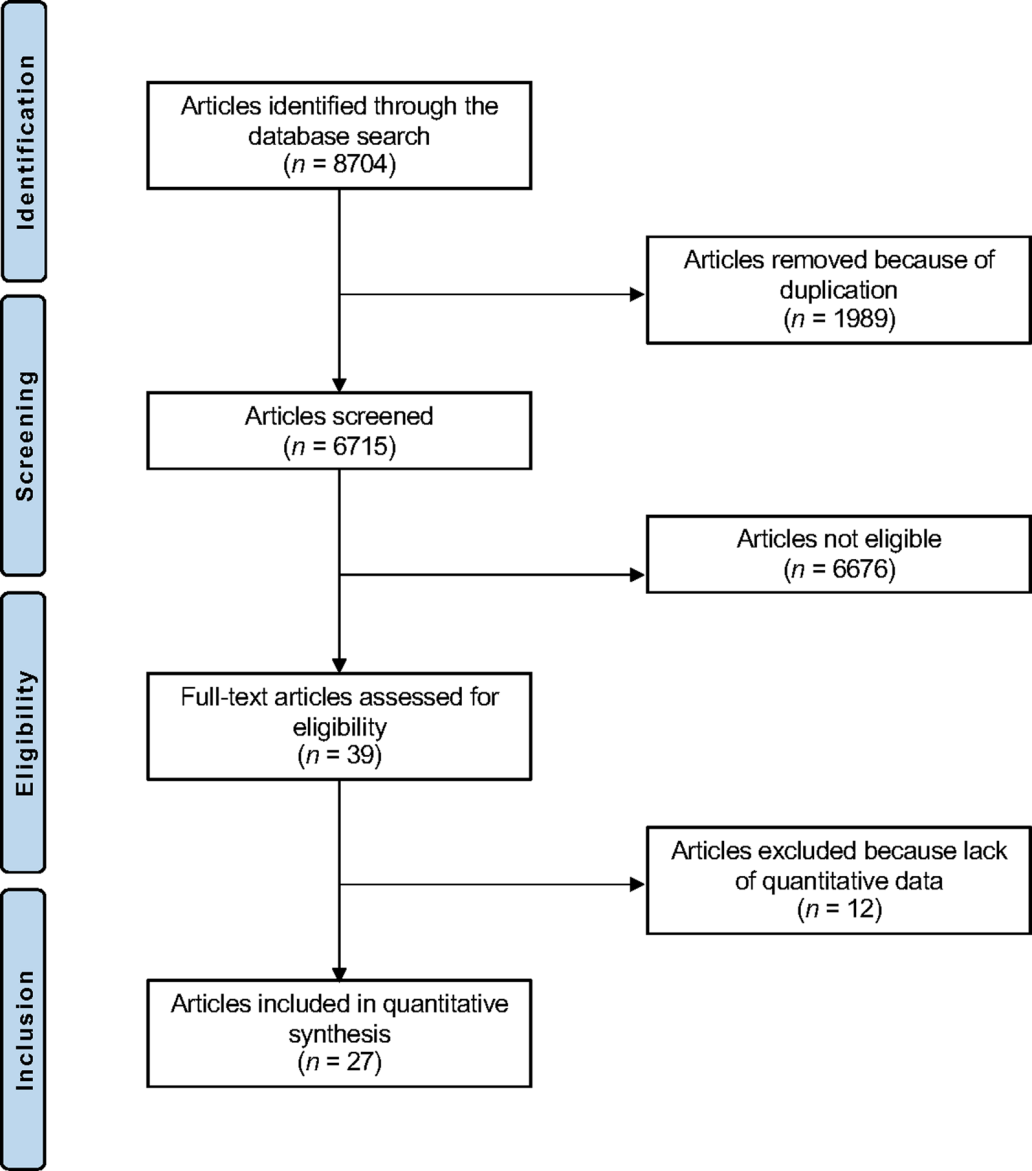
PRISMA flowchart of the literature search

The literature search resulted in 8704 articles. Of them, 1989 were excluded as they were duplicates. A further 6688 articles were excluded with reason: no matching the topic of interest (*N* = 6357), study type not adequate (*N* = 301), with uncertain results or diagnosis (*N* = 15), not reporting any data on the outcomes of interests (*N* = 12), language limitation (*N* = 3). Finally, 27 case reports were included in the present systematic review. The results of the literature search are shown in Fig. 1.

### Study characteristics and synthesis of results

Data from 54 patients were retrieved, with a mean age of 49.8 ± 14.5 years. 54% (29 of 54 patients) were women. Most studies were conducted in India, Pakistan, Italy and United Kingdom. Two cases per country were reported in Japan, Libanon, Saudi Arabia, Turkey, USA, whereas Denmark Iran Germany, Kazakhstan, Portugal, and Singapore reported only one case each. The generalities and demographic of the included studies are reported in Table 1.

**Table 1:** Generalities and patient baseline of the included studies

Author and year	Journal	Country	Patients (*n*)	Mean age
Baimukhamedov et al. 2021 [2]	*Lancet Rheumatol*	Kazakhstan	1	67
Basheikh et al. 2022 [4]	*Cureus*	Saudi Arabia	1	43
Cincinelli et al. 2021 [10]	*Medicine*	Italy	1	27
Coath et al. 2020 [11]	*Rheumatology*	United Kingdom	1	53
Danssaert et al. 2020 [13]	*Cureus*	USA	1	37
De Stefano et al. 2020 [14]	*Ann Rheum Dis*	Italy	1	30 s
Di Carlo et al. 2021 [15]	*Clin Exp Rheumatol*	Italy	1	55
Fragata et al. 2020 [19]	*Acta Reumatol Port*	Portugal	1	41
Gasparotto et al. 2022 [22]	*Clin Rheumatol*	Italy	1	60
Ghauri et al. 2020 [41]	*Int J Clinl Rheumatol*	Pakistan	1	34
Gibson et al. 2020 [23]	*Rheumatol Adv Pract*	United Kingdom	1	37
Hasbani et al. 2021 [17]	*Reumatismo*	Libanon	2	41
Hønge et al. 2021 [26]	*Bmj Case Reports*	Denmark	1	53
Jali et al. 2020 [30]	*Cureus*	Saudi Arabia	1	39
Kocyigit et al. 2021 [35]	*Rheumatol Int*	Turkey	1	53
Liew et al. 2020 [37]	*J Clin Rheumatol*	Singapore	1	47
Mukarram et al. 2021 [42]	*Case Rep Rheumatol*	Pakistan	5	39.4
Ono et al. 2020 [43]	*RMD Open*	Japan	1	50 s
Pal et al. 2022 [46]	*Reumatol Clin*	India	23	42.8
Parisi et al. 2020 [49]	*Lancet Rheumatol*	Italy	1	58
Saikali et al. 2021 [51]	*Immun Inflamm Dis*	USA	1	21
Saricaoglu et al. 2020 [53]	*J Med Virol*	Turkey	1	73
Schenker et al. 2020 [54]	*Rheumatology*	Germany	1	65
Shokraee et al. 2021 [57]	*Trop Dis Travel Med Vaccines*	Iran	1	58
Sureja et al. 2021 [58]	*Rheumatol Adv Pract*	India	1	27
Waller et al. 2020 [61]	*Rheumatol Adv Pract*	United Kingdom	1	16
Yokogawa et al. 2020 [63]	*Ann Rheum Dis*	Japan	1	57

*RCT*—Randomised controlled trial

Between patient severity of COVID-19 was heterogeneous. Most patients were affected by mild COVID-19 and did not require hospitalisation. Few studies observed the occurrence of reactive arthritis in inpatients who experienced more severe COVID-19 [22, 26, 35, 43, 53, 54, 57, 58, 63] and required intensive cares and/or mechanical ventilation [22, 43]. The mean time span between COVID-19 infection and the occurrence of reactive arthritis symptoms was 22.3 ± 10.7 days. An overview of the pharmacological management, body location, and main findings of the included studies is shown in Table 2.

**Table 2:** Overview of the pharmacological management, body location, and main findings of the included studies

Author and year	Treatment OA	Location OA	Main results of the study
Baimukhamedov et al. 2021 [2]	15 mg methotrexate weekly, 8 mg methylprednisolone daily	Knee, hand	Presence of autoantibodies after COVID-19 infection might suggest that this virus might also act as a trigger of reactive arthritis
Basheikh et al. 2022 [4]	1600 mg ibuprofen daily, 25 mg prednisolone PO daily (5 days)	Spine	The patient responded to 5-days steroids and NSAIDs. The early recognition and treatment of reactive arthritis ensured a favourable outcome
Cincinelli et al. 2021 [10]	10 mg prednisolone PO daily	MCP	The patient responded to steroids. Like many other viral diseases, COVID-19 can play as a causative agent or as a trigger for inflammatory arthritis development in predisposed individuals
Coath et al. 2020 [11]	120 mg IM methylprednisolone daily, 75 mg diclofenac daily,	Spine, chest wall	There was a definite temporal relationship between COVID-19 infection and the onset of axial disease, in a timeframe that would typically be expected for reactive arthritis
Danssaert et al. 2020 [13]	Dilaudid IM and PO, oxycodone PO, neurontin daily	Hand	It is possible that symptoms could have been unrelated to COVID-19, but the extensive workup in the hospital did not reveal another potential cause
De Stefano et al. 2020 [14]		Elbow	A state of virus induced transient immunosuppression may predispose to reactive arthritis even in the absence of a genetic background
Di Carlo et al. 2021 [15]	4 mg methylprednisolone daily	Ankle	COVID-19 was considered the infectious trigger of reactive arthritis diagnosed. This conclusion was also supported by the time interval of few weeks between reactive arthritis onset and COVID-19
Fragata et al. 2020 [19]	1200 mg ibuprofen daily; 5 mg PO prednisolone (5 days)	PIP, DIP, MCP	Reactive viral arthritis might be a late complication of COVID-19
Gasparotto et al. 2022 [22]	600 mg ibuprofen (30 days)	Knee, ankle	Molecular mimicry might be the basic immunological mechanism responsible for the onset of COVID-19-related arthritis based on the current knowledge of COVID-19 and on the known pathogenetic mechanism of viral-induced arthritis
Ghauri et al. 2020 [41]	IA steroids, NSAIDs	Knee	Physicians should have a high index of suspicion while treating any post infectious COVID-19 patient who presents with joint pain or arthralgia
Gibson et al. 2020 [23]	20 mg prednisolone OS daily, NSAIDs	ankle, wrist, PIP, shoulder, elbow, knee	A self-limiting episode of inflammatory arthritis may occur following COVID-19 infection
Hasbani et al. 2021 [17]	1 g naproxen daily, 40 mg prednisolone daily; 1 g sulphasalazine daily	Sacroiliac, ankle, elbow, wrist	COVID-19 can induce reactive arthritis even in not-predisposed population
Hønge et al. 2021 [26]	1200 mg OS ibuprofen daily; 25 mg OS prednisolone daily (6 days)	Knee, ankle	The condition improved markedly after a few days of NSAIDs and prednisolone administration
Jali et al. 2020 [30]	Celecoxib (2 weeks)	DIP, PIP	Rheumatologists should consider Reactive arthritis as a possible complication of COVID-19, after an appropriate differential diagnostic
Kocyigit et al. 2021 [35]	150 mg diclofenac daily (6 weeks)	Knee	Comprehensive clinical and laboratory investigations, synovial fluid analysis, and close follow-up of the patient are essential
Liew et al. 2020 [37]	Etoricoxib, triamcinolone IA	Knee	The high CT value of his COVID-19 swab on admission indicated low copies of viral RNA, suggesting that the patient was late in the course of COVID-19 when he developed reactive arthritis
Mukarram et al. 2021 [42]	10 mg prednisone daily, etoricoxib, 20 mg leflunomide daily, 400 mg hydroxychloroquine daily	PIPs, MTPs, MCPs, wrist, ankle	There is not sufficient data to indicate that people develop autoimmune inflammatory arthritis after being affected by COVID-19. Therefore, this case series adds a substantial amount of evidence to this hypothesis
Ono et al. 2020 [43]	NSAIDs, steroids IA	Ankle	reactive arthritis should be considered in patients with acute arthritis after COVID-19
Pal et al. 2022 [46]	NSAIDs, hydroxychloroquine, methotrexate, steroids	Knee, ankle, wrists, MTPs, MCPs, shoulder, hip, spine	Axial symptoms and enthesitis were often coexistent. Treatment with NSAIDs and intra-articular steroids was effective. Whether COVID-19 was the definitive aetiology of the arthritis is yet to be proven
Parisi et al. 2020 [49]	1200 mg ibuprofen daily	Ankle	First case of arthritis in a COVID-19 patient in Europe
Saikali et al. 2021 [51]	TNF $\alpha$ inhibitor, certolizumab	sacroiliac, spine, knee	The importance of autoimmune and autoinflammatory diseases being triggered by COVID-19
Saricaoglu et al. 2020 [53]	NSAIDs	MTP, PIP, DIP	reactive arthritis as complication of COVID‐19 will raise the awareness of the physicians
Schenker et al. 2020 [54]	Prednisolone	Knee, ankle, wrist	The timely relation, presence of HLA-B27 as well as the strong anti-COVID-19 IgG antibody response support the concept that COVID-19 induced an autoimmune response that led to reactive arthritis and vasculitis
Shokraee et al. 2021 [57]	200 mg indomethacin daily, 80 mg prednisolone IM	Hip, sacroiliac	COVID-19 can lead to autoimmune reactions, including reactive arthritis
Sureja et al. 2021 [58]	NSAIDs, opioids	Knee, ankle, feet, wrist, MCP, PIP	The classical clinical picture, a preceding infection, absence of other autoantibodies, absence of autoimmunity in the family and response to NSAID, supported the diagnosis of reactive arthritis following COVID-19
Waller et al. 2020 [61]		MCP, wrist, shoulder, hip, knee	Classic post viral reactive arthritis has been seen following COVID-19. A registry to collect information on de novo autoimmune presentations would be highly informative
Yokogawa et al. 2020 [63]		Wrist, shoulder, knees	COVID-19 viraemia was absent in the case reported, which shared clinical features with reactive arthritis due to hepatitis B and C virus, parvovirus or alphavirus, such as chikungunya

*MCP*—Metacarpophalangeal, *DIP*—Distal interphalangeal, *PIP*—Proximal interphalangeal, *TNF*—Tumour necrosis factor, *NSAIDs*—Non-steroidal anti-inflammatory drugs, *PO*—Per OS, *IM*—Intra-muscular, *IA*—Intra-articular, *HLA*—Human Leukocyte Antigen

## Discussion

Low quality evidence suggests that COVID-19 infection can act as a causative agent or as a trigger for the development of reactive arthritis even in patients who did not show the antibodies of rheumatological disorders. The diagnosis was made by exclusion: all patients shared a previous COVID-19 infection approximatively 22 days prior of the symptoms. This length is similar to what described in other reactive arthritis, which is approximately between few days to 4 weeks after an infection [25, 60]. All cases resolved within few days in all studies considered. At last follow-up, all patients were minimally symptomatic or asymptomatic, and no additional therapy or attentions were required by any patient.

The pharmacological management was heterogeneous. The therapy lasted five to 42 days. NSAIDs should be used as first line and are recommended in treatment of peripheral arthritis [16, 21, 36]. Indeed, among the included studies, NSAIDS, including etoricoxib, celecoxib, indomethacin, naproxen, and ibuprofen were the most commonly administered compounds [11, 17, 19, 22, 23, 26, 30, 35, 37, 42, 43, 49, 53, 57, 58]. The most common orally administered steroids were 10–80 mg prednisolone [4, 10, 17, 19, 23, 26, 42, 54, 57], and 4–8 mg of methylprednisolone [2, 15]. Two authors performed intra-articular injections of steroids (e.g. triamcinolone) [41, 43], and one administered 120 mg methylprednisolone intra-muscular daily [11]. The intra-articular use of steroids in mono- or oligoarthritis demonstrated efficacy in previous studies [47, 59]. Opioids (e.g. oxycodone) were also administered orally [13, 58]. Disease-modifying antirheumatic drugs (DMARDs) such as sulfasalazine can also be used [55]. Recently, biologics have also been introduced in the management of ReA [64]. Among the included studies, even if less commonly administered, intra-muscular and oral dilaudid [13], neurontin [13], TNF $\alpha$ inhibitor [51], certolizumab [51], 1 g sulfasalazine [17], 20 mg leflunomide [42], and 200–400 mg hydroxychloroquine daily [42, 46] were also used. One author also administered 15 mg methotrexate weekly [2, 46].

At plain radiography, an effusion was reported in two patients in plain radiographies [26, 37]. Most patients did not evidence any bony involvement [4, 17, 22, 30, 35, 41, 43, 53], whereas in two cases mild sclerotic changes were noted [37, 51]. At magnetic resonance imaging, mild effusion, with aspecific soft tissue swelling and subcutaneous and bone marrow oedema were evidenced [11, 13, 17, 41, 51, 57]. At joint sonography, synovitis and articular effusion were reported [15, 22, 35, 41, 57] [49]. Four studies documented the involvement of surrounding tendons [13, 15, 17, 46]. Most of patients who had reactive arthritis at the ankle evidenced enthesitis of the Achilles tendon [23, 26, 43, 46, 49]. A mild inflammatory hypercellularity [22, 43] of synovial aspirate, with no evidence of crystal deposition [22, 26, 35, 37, 63], was found in most patients at polarized light microscopic examination.

The location of reactive arthritis was heterogeneous. Similar to other spondiloarthropathies, reactive arthritis has a tendency to affect the lower extremities and can present with enthesitis and dactylitis [21, 32, 56]. Indeed, the knee was the most common location of the pain, followed by the hand and the ankle. Less common locations were the spine and sacroiliac joint, foot, wrist and the hip. Articular involvement in reactive arthritis can be either monoarthritis or oligoarthritis [21, 32]. Hence, most patients had an oligoarthritis [2, 11, 17, 19, 22, 23, 26, 30, 42, 43, 46, 49, 51, 53, 54, 57, 58, 61, 63], and some a monoarticular involvement [10, 13–15, 35, 37, 41]. With regards to the body location (right and left) of reactive arthritis, the distribution of the involved joints was bilateral [2, 17, 19, 23, 30, 42, 43, 46, 51, 54, 58, 61], or involved only one side of the body [10, 13–15, 22, 26, 35, 37, 41, 49, 57]. There was no symmetrical distribution in most bilateral cases. At clinical examination, warmth, redness, swelling, and decreased range of movement was reported by most authors [10, 13, 15, 22, 23, 30, 37, 41, 53, 58, 61, 63]. Most patients had no associated extra-articular manifestations. A few patients reported associated psoriasis [14, 46], bilateral conjunctivitis [4, 46], myalgia [46], balanitis [4, 37], and symmetrical vasculitis at the calf [54, 61] and trunk [61]. Additional symptoms, including fever, cough, nausea, diarrhoea, and dysgeusia were inconstant.

Serological results were also heterogeneous. C-reactive protein was positive in some patients [2, 4, 11, 15, 17, 23, 26, 35, 46, 49, 53, 54, 63], but not in other patients [13, 19, 22, 30, 43]. Almost all authors investigated the presence of antibodies of rheumatological disorders, and their presence was inconstant. Of 54 patients, 14 (26%) had antibodies. One patient presented anti-carbamylated protein antibody (ACPA) [2], one presented antinuclear antibodies (ANA) [13], two rheumatoid factor (RF) [2, 58], and 11 positive HLA-B27 [11, 17, 46, 54]. These data are consistent with the current evidence, which estimated that 30% to 50% of patients with reactive arthritis have associated positive HLA-B27 [8, 24], and that the disease is five times more prevalent in patients who are HLA-B27 positive compared to the general population [12, 18, 62]. Most patients have no history of autoimmunity, inflammatory bowel disease or travel history, and had been prescribed no new medication.

The results of the present systematic review must be considered in the light of some important limitations. All included studies are case reports, and the results are subjected to several confounders. Case reports lack of generalisability to larger populations of patients, with high risk of over- or mis-interpretation when generalized to clinical practice. In conclusion, the results of the present study lack of generalisability to larger populations of patients, with high risk of over- or mis-interpretation when generalized to clinical practice. Between studies heterogeneity in diagnosis, treatment, examinations, and patient’s ethnicity were evident. Established diagnostic or classification criteria for reactive arthritis are missing; the American College of Rheumatology (ACR) proposed general principles in a 1999 workshop [56]. A registry to collect information on de novo autoimmune presentations would be highly informative. Deeper understanding of the immune mechanism related to COVID-19 may useful opportunity to further investigate the immunopathogenic mechanisms capable of promoting or contrasting the development of specific rheumatic diseases. Close monitoring on the prevalence and expressiveness of rheumatic disorders is required.

## Conclusion

Poor evidence suggests that COVID-19 could target the musculoskeletal system causing reactive arthritis at its post infectious stage. COVID-19 can play as a causative agent or as a trigger for reactive arthritis development even in patients without presence of antibodies of rheumatoid disorders. The treating physician or rheumatologist should have a high index of suspicion while treating any post infectious COVID-19 patient with arthralgia.

## Data Availability

The datasets generated during and/or analysed during the current study are available throughout the manuscript.
